# Efficient Violence Detection in Surveillance

**DOI:** 10.3390/s22062216

**Published:** 2022-03-13

**Authors:** Romas Vijeikis, Vidas Raudonis, Gintaras Dervinis

**Affiliations:** Department of Automation, Faculty of Electrical and Electronic Engineering, Kaunas University of Technology, 51367 Kaunas, Lithuania; vidas.raudonis@ktu.lt (V.R.); gintaras.dervinis@ktu.lt (G.D.)

**Keywords:** violence detection, violent behavior, intelligent video surveillance, computer vision, U-Net, LSTM, deep learning

## Abstract

Intelligent video surveillance systems are rapidly being introduced to public places. The adoption of computer vision and machine learning techniques enables various applications for collected video features; one of the major is safety monitoring. The efficacy of violent event detection is measured by the efficiency and accuracy of violent event detection. In this paper, we present a novel architecture for violence detection from video surveillance cameras. Our proposed model is a spatial feature extracting a U-Net-like network that uses MobileNet V2 as an encoder followed by LSTM for temporal feature extraction and classification. The proposed model is computationally light and still achieves good results—experiments showed that an average accuracy is 0.82 ± 2% and average precision is 0.81 ± 3% using a complex real-world security camera footage dataset based on RWF-2000.

## 1. Introduction

Video camera-based surveillance is an extremely efficient and economical solution to ensure the security of valuables, property, buildings, and people. Video cameras are used for remote monitoring of complex environments in different weather conditions, which involve constantly changing illumination, temperature, and visibility. Manufacturers of the surveillance equipment are addressing these issues by providing modern cameras, which have higher resolution, built-in heating elements, automatic image enhancement in low-light conditions, and encoding and encrypting technologies. Modern internet protocol (IP) cameras have 4K resolution capabilities, which are 27 times larger than conventional analogue cameras. The increase in image resolution demands wider bandwidth for the data streaming. The required total bandwidth depends on the number of cameras, image resolution, frame rate, and the rate of compression. Continuous data streaming for surveillance network cameras results in bandwidth issues, loss of connection, and other network-related problems. Single video frame needs from 30 to 160 Kbytes and with a higher frame rate per second, every camera could require up to 10 Mbps. High bandwidth usually costs more, because of the expense for a strong network connection. This limiting factor raises challenges for the usage of IP-based video surveillance. Moreover, networks are becoming vulnerable and bandwidth issues have come to the forefront because of the rising need for cloud computing.

Security surveillance systems based on IP and closed-circuit television (CCTV) cameras are already ubiquitous in public spaces. The demand for video surveillance systems in public places are increasing for the purpose of ensuring security in different application areas, such as retail, enterprises, banks, other financial institutions, and other public places. Video surveillance systems can help to solve and prevent many criminal activities. However, video surveillance is often viewed as a violation of privacy by the majority of people. There are concerns about how video recordings might be used or misused especially when modern image processing technologies are applied. Privacy protection laws have been introduced in many countries and regions, such as General Data Protection Regulation (GDPR) in Europe and the Federal Information Security Management Act (FISMA) in the USA. These laws demand businesses and other users of surveillance systems to take greater responsibility for captured data.

The authors of this research work propose to use a dedicated AI method that is efficient in the sense of energy and memory consumption and capable of running on embedded edge devices. The application of the AI method addresses several issues. First, a specific dedicated task, such as face recognition and or in this case violence detection, can be done locally on the edge device. In this way, the requirements for the bandwidth can be reduced to only passing a message containing relevant data, because only descriptive features and important (informative) frames are transmitted in the network. There is no need to transfer video data into the cloud for later image processing and recognition. Secondly, all privacy-related data can be filtered by leaving only the video data that directly relates to a certain crime or accident. The authors of the research work are focusing on developing a deep learning-based algorithm that is capable of detecting violence from the surveillance video stream. The main value of such intelligent surveillance interpreting scenes on the network edge is the reduction of the reaction time in case of harm to public health and safety. The proposed solution could help by attracting the attention of the human operator to the screen, which is highlighted by the algorithm and accordingly the operator would react faster. Recent developments of computer vision and machine learning techniques enable intelligent video surveillance (IVS). Systems enhance the perception and reduce the reaction time of human operators by autonomously extracting the relevant information about the scene.

The paper is organized as follows: Section 2 reviews some research works that describe the most common methods to detect violence from video features, Section 3 gives a description of the proposed model, Section 4 contains experiments and results, Section 5 contains discussion part, and Section 6 summarizes the article.

## 2. Related Works

The need for violence detection in the video cannot be overemphasized as its application relevance cuts across maximizing the security of citizens, mitigating against violent behavior in children, threat detections, reduction in response time of the first responders, etc. Thus, the need for analyzing violent activities in humans using surveillance videos is extremely valuable. Therefore, this section discusses the various methods and techniques used in previous research, focusing on the recognition of violent activity from video surveillance. Recently, the adoption of various artificial intelligence methods in computer vision has helped greatly in the identification of violent activities from video datasets. To further discuss the research progress in the detection of violent activities, we categorized this section into two parts: violence detection based on machine learning techniques and violence detection based on deep learning techniques.

### 2.1. Violence Detection Based on Machine Learning Techniques

Penet et al. [1] investigated the different Bayesian network learning algorithms using temporal and multimodal information for a violent shot detection system. The experimental analysis was carried out using the MediaEval 2011 Affect Task corpus and the zero-crossing rate (ZCR) feature extraction model was employed. The authors implemented two-score learning algorithms and the overall results gave a false alarm rate of 50% and 3% missed-detection. Another interesting study was presented by Deniz et al. [2] where extreme acceleration patterns were received by performing random transforms against the power spectrum of consecutive video frames used as the main feature of the model to recognize violence. Experiments show 12% higher accuracy in comparison to state-of-the-art techniques for violent event recognition.

Nievas et al. [3] proposed to use the bag-of-words (BoW) approach and two motion descriptors, space–time interest points (STIP) and motion scale invariant feature transform (MoSIFT) for fight detection. The experimental results using the BoW method were able to detect fights with 90% accuracy. Another application of the advanced feature extraction model was implemented by De Souza et al. [4] and a comparison of the performance of two feature extraction models based on SIFT and STIP using the same datasets. The experimental results showed that the overall performance of STIP outperforms the SIFT technique. A similar study was also carried out by Xu et al. [5], which presented the combination of support vector machine (SVM) with radial basis function (RBF) kernel for the detection of violence. The study applied feature extraction based on MoSIFT or STIP for extracting relevant shape and motion patterns of activity, thus improving violence detection.

A violent flow (VF) variation for violence detection based on the combination of SVM and Horn–Schunck optical flow algorithm was proposed by Arceda et al. [6]. Das et al. [7] applied histogram-oriented gradient (HOG) to extract lower level features from the video clips. The authors applied six machine learning classifiers, which include SVM, logistic regression, random forest, linear discriminant analysis (LDA), natıve Bayes and K-nearest neighbors (KNN) for the classification of violent activities in surveillance scenarios. The proposed model using a random forest classifier achieves 86%. The performance on the benchmark dataset achieves very high accuracy with significant improvement from previously proposed methods.

Gracia et al. [8] compared the performance of three machine learning algorithms using SVM, AdaBoost, and random forests classifiers for the detection of a fast fight based on three video datasets. The experimental results showed that the proposed method could not outperform the best state-of-the-art methods, with an accuracy ranging from 70% to 98%. Another study by Fu et al. [9] presented a low computation method in the automatic detection of fights. The study was based on two feature extraction models that include the optical flow and the BoW approaches. The experiment’s results with respect to accuracy showed that the proposed method exceeds other approaches using the combination of the BoW mechanism on machine learning algorithms. Senst et al. [10] proposed another method for violence detection based on nonlinear dynamic systems. The authors integrated Lagrangian theory into the SIFT and BoW methods. The performance of the model showed a tradeoff between efficiency and computational complexity in the context of violence detection.

Another technique for violence detection was presented by Febin et al. [11]. The method, using motion boundary SIFT (MoBSIFT) and movement filtering modules, was tested with two publicly available datasets. To reduce the time complexity posed by the MoBSIFT technique, the authors changed the optical flow estimation to dense optical flow estimation for the entire frame once and thus completely removed the difference of Gaussian (DOG) pyramid-based flow estimation. Another study by Zhang et al. [12] applied an optical flow method and the Gaussian model to train an SVM classifier. An extended study was conducted by Mahmoodi and Salajeghe [13], which applied a machine learning classifier on a new features extraction approach based on the histogram of optical flow magnitude and orientation.

Other algorithms proposed by previous researchers for the detection of violent scenes include the adoption of Kohonen’s self-organizing map (SOM) (Clarin et al. [14]), improved Fisher vectors (IFV) by Bilinski and Bremond [15], and a novel framework based on dense trajectories (DT) combined with MPEG flow video descriptor presented by Cai et al. [16].

### 2.2. Violence Detection Based on Deep Learning Techniques

Recent studies by Ullah et al. [17] proposed a deep learning three-stage end-to-end violence recognition framework based on spatiotemporal features. The authors optimized the 3-dimensional convolution neural network (3D CNN) model by converting the trained model to intermediate representation employing an open visual inference and neural networks for the automatic violent event detection. The analysis was performed on three publicly available datasets and the experimental performance shows that the highest accuracy was achieved with the Hockey Fights dataset. Patel [18] introduced a hybrid CNN based on ResNet 50 architecture and long short-term memory (LSTM) for violence detection on three video datasets, namely the Hockey Fights, Violent Flows, and Movie Fights. The experimental result shows that the proposed hybrid method achieved better accuracy in comparison with other deep learning architectures such as InceptionV3 and Visual Geometry Group containing 19 layers (VGG19).

Similarly, Baba et al. [19] presented a lightweight CNN model such as MobileNet and SqueezeNet model on two publicly available datasets, namely the BEHAVE and ARENA datasets. In order to optimize the performance model, a time domain filter was applied with the aim of differentiating between violent scenes in a video within a certain timeframe. The overall experimental result was able to achieve an accuracy of 77.9% with no misclassification of violent class and a high false-positive rate of 26.76% of the nonfight clips. One of the shortcomings of the proposed method as stated by the authors is the fact that the model cannot detect violence in the crowd.

Use of deep learning for violence detection began in 2012 when Krizhevsky et al. [20] proposed a new approach, which they called ImageNet—a model that improved the error rate to 16.4% (for comparison, at the time the best error rate was 26.1%). Zeiler and Fergus [21] explained factors that are accountable for this advancement and other benefits of CNN. This section describes some most popular deep learning-based methods for violence recognition from videos.

CNN and LSTM combination for violence detection in video features was created recently. CNN is used for feature extraction at the frame level. Collected features are classified as violent or nonviolent by utilizing a variant of LSTM in the works by Sudhakaran and Lanz [22], Soliman et al. [23], Letchmunan et al. [24], and Sumon et al. [25]. By combining CNN and LSTM, spatiotemporal features existing in the video are localized, which allows for local motion analysis. Several pretrained CNN models, namely VGG16 by Soliman et al. [23], Sumon et al. [25], VGG19 by Letchmunan et al. [24], Sumon et al. [25] and ResNet50 by Sumon et al. [25] were used to separate the spatial features, prior to their classification as violent or nonviolent events.

Sudhakaran and Lanz [22] additionally suggested using the difference of neighborly frames as an input; this model could encode differences that are shown in videos. Validation of the algorithm was executed with three datasets: Hockey Fights, Movie Fights, and Violent Flows. Experiments showed that the proposed model is more accurate than most popular state-of-the-art methods and reached 97.1%. ConvLSTM is also proposed by Shi et al. [26], where it expands the LSTM model to contain convolutional structure in both input-to-state and state-to-state transitions. Experiments showed that ConvLSTM is able to catch spatiotemporal correlations precisely and demonstrate encouraging results. Usually, methods that detect violence deeply depend on complicated handcrafted features and algorithm teaching methods. On the other hand, the deep learning-based model can operate directly and perform automatic feature extraction. Ding et al. [27] proposed a new technique where motion information is obtained by the input by applying 3D CNN and does not require prior information for violence detection. Experiments showed that the proposed method achieves better results in terms of accuracy and does not rely on prior information. Results presented in Tran et al. [28] shows that 3D CNN can precisely determine spatiotemporal relation and, in addition, can exceed the ConvLSTM method. 3D CNN consists of four dimensions: time, height, width, and colors. Results presented in Tran et al. [28] show that 3D CNN is simpler, faster, and more straightforward for training when compared to the ConvLSTM method. Furthermore, with sufficient training information, it is one of the best frameworks for action recognition.

Hanson et al. [29] proposed a model which is separated into three parts: spatial encoders, temporal encoders, and classifiers. The authors use already existing ConvLTSM architectures that are supplemented with bidirectional temporal encodings. The model presented is called a one-stream model, as it uses only one format for input; nevertheless, there are works that presented models that use both formats for their input. These models are called convolutional multistream models, where the type of each video stream is analyzed as well. Zhou et al. [30] proposed a multistream model named FightNet. The model can recognize three input types: RGB, optical flow, and acceleration images. The model divides the input into components and obtains the stream for each component feature map. Eventually, each feature map is combined and the average score of each component of the video is calculated as an output.

A summary of some related literature is presented in Table 1.

### 2.3. Limitations of Current State-of-the-Art Methods

Based on some of the overall findings deduced from our extensive literature review, we can conclude that some of the existing methodology used by previous research still requires more advanced methods with the aim of combating existing limitations such as insufficient data frames from video clips, challenges with increasing false alarm rate, and high computational methods based on time complexity for real-time detection.

The insufficient data frames from the video clips are remedied in this work by performing the analytics on the edge device having direct access to the video camera, therefore avoiding the video compression and frame skipping used to reduce the network bandwidth. Therefore, this study aims to apply the proposed method for effective detection of violence from surveillance videos by focusing on the reduced computational load and reduction of the false-positive alarm rate, thus improving security and reliability. False-positive alarm rate is inversely proportional to the precision, for which we were optimizing.

Violence detection in surveillance video is a complex task because of many factors, because of violence unpredictability, varying environmental conditions, and image noise. Therefore, image-enhancing methods proposed by Wei et al. [36] can be applied to address image quality issues and further improve performance.

## 3. Proposed Model

The main goal of the proposed model is to maintain the performance comparable to state-of-the-art violence detection models while reducing the computational complexity for the deployment on low power (<20 W) edge devices.

The proposed algorithm is mainly divided into three stages:

1. Spatial feature extractor (time-distributed U-Net);
2. Temporal feature extractor;
3. Classifier.

**Figure 1:**
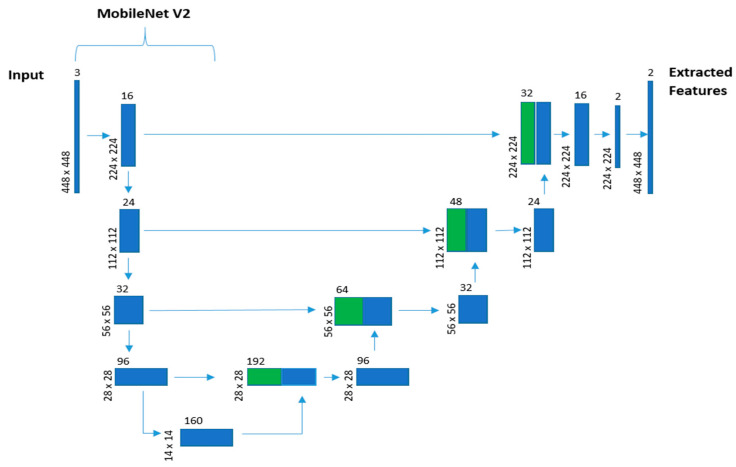
U-Net and MobileNet V2 network model. Green shows copies of the encoder (MobileNet V2) features maps concatenated to the decoder feature maps.

The model receives batches of 30 frames of the security camera videos corresponding to 1 s length slices of the footage. Once the video frame is received by the model, the first stage of the model is a spatial feature extraction using a U-Net-like network. This network uses MobileNet V2 as an encoder in the U-Net-like architecture used to do the static single frame spatial feature extraction in a sequential-time-distributed manner, as shown in Figure 1.

**Figure 2:**
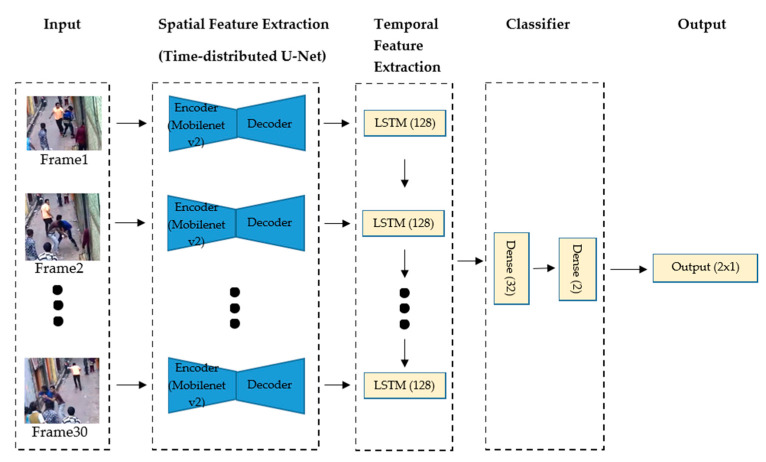
Proposed model architecture.

Thus, a new queue of frame features is passed to the second stage for temporal feature extraction. LSTM is employed to obtain the sequential information between consecutive slices of the video. Having all this information, the two-layer classifier, based on dense layers, annotates events as violent or nonviolent. The architecture of the proposed model is visualized in Figure 2.

**Figure 3:**
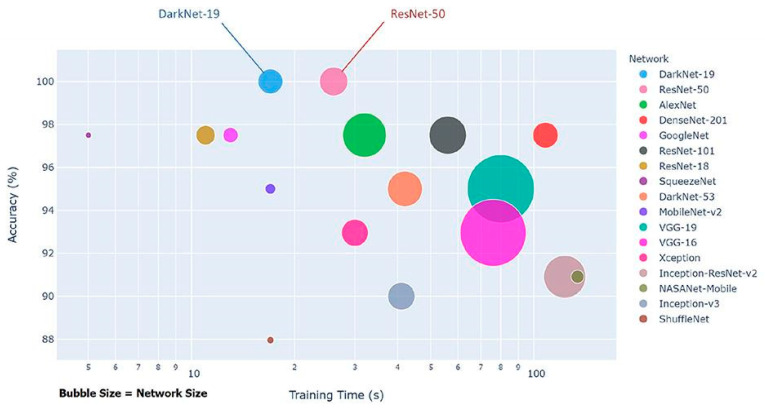
MobileNet V2 comparison with other state-of-the-art classifiers in terms of accuracy (Elgendi et al. [37]).

As a classifier for the model, we chose a simple state-of-the-art classifier for spatial feature extraction, MobileNet V2. As can be seen in Figure 3, MobileNet V2 achieves high accuracy with a significantly smaller network size.

The remaining network is a simple LSTM classifier needed for temporal feature classification to detect fight movements. Table 2 shows the parameters of the model.

Since the model realizes the function of binary classification for videos, the loss function used in this paper is the binary cross-entropy (BCE) loss function. The loss function used for model fitting follows:

(1) $$\mathrm{BCE}=-\frac{1}{\mathrm{output}\;\mathrm{size}}\overset{\mathrm{output}\;\mathrm{size}}{\underset{i=1}{\sum }}y_{i}\cdot \mathrm{loglog}\;\hat{y_{1}}+\left(1-y_{i}\right)\cdot \mathrm{loglog}\;\left(1-\hat{y_{1}}\right)$$

where $\hat{y_{1}}$ is the *i*-th scalar value in the model output, $y_{i}$ is the corresponding target value, and output size is the number of scalar values in the model output.

### Motivation of Using MobileNet V2

For our model, we chose to use pretrained MobileNet V2 [38] architecture as the encoder component in the spatial features extracting the U-Net-like model because it is:

1. Computationally light while maintaining the same (or very close) performance as compared to other state-of-the-art convolutional classifiers;
2. More suitable for real-time operations using bandwidth-restricted hardware architecture because of lower memory requirements.

In short, MobileNet V2 is smaller in terms of the number of computations and learned parameters while still maintaining good accuracy.

We integrated MobileNet V2 into the U-Net-like feature extractor, as shown in Figure 1. The model uses an encoder that is pretrained against the Imagenet dataset. This improves the training effectiveness due to unlabeled spatial data in frames. Most of the violence-indicating information is temporal, visible from motion and not static frames. In addition, the environments of the scenes vary greatly in the security camera footage. Providing an effective and efficient pretrained general spatial-feature extractor reduces the training complexity to correlating the change of these features through time to the instances of violent behaviors.

## 4. Experiments and Results

This section describes materials used for experiments and explains the results of experiments.

### 4.1. Dataset

There are various datasets available; however, in this work we use three datasets:

1. RWF-2000 dataset for violence detection by Cheng et al. [39];
2. Movie Fights dataset by Nievas et al. [3];
3. Hockey Fights dataset by Nievas et al. [3].

**Figure 4:**
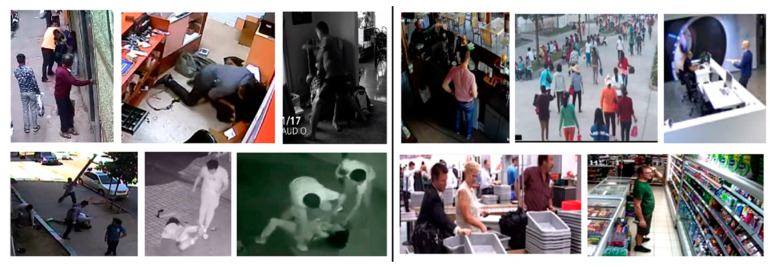
Examples of violent (on the **left**) and nonviolent (on the **right**) scenes from the RWF-2000 dataset.

**Figure 5:**
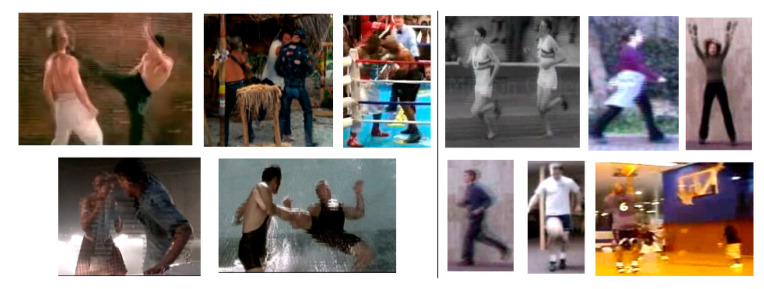
Examples of violent (on the **left**) and nonviolent (on the **right**) scenes from the Movie Fights dataset.

**Figure 6:**
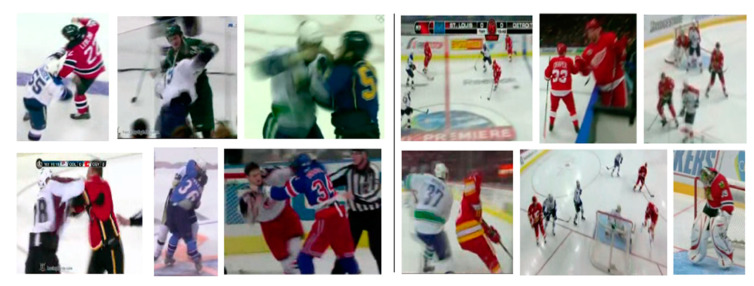
Examples of violent (on the **left**) and nonviolent (on the **right**) scenes from the Hockey Fights dataset.

Figure 4, Figure 5 and Figure 6 show examples of violent and nonviolent scenes from the RWF-2000, Movies Fights, and Hockey Fights datasets, respectively.

The RWF-2000 dataset contains 2000 video samples: 1000 violent and 1000 nonviolent, which were taken by surveillance cameras in different real-life situations. The RWF-2000 dataset was modified; the videos that had violent scenes longer than 1 s in the video were trimmed so the violent event starts at the beginning of the video. Even though the description of the dataset stated that all videos are 30 fps, there were 400 videos in total that contained multiple frames resulting in affecting far lower frame rates; for example, some videos had five or more frames which were duplicated to achieve 30 fps. These videos were removed from the dataset, only complete videos were separated from the dataset. In the end, we had 1600 videos to work with. The 1600-video dataset was shuffled and divided into 960 videos for testing, 320 for validation, and another 320 for testing.

The Movie Fights dataset contains 200 video samples: 100 for violent and 100 for nonviolent scenarios. The Hockey Fights dataset contains 1000 video samples: 500 for violent and 500 for nonviolent scenarios.

All datasets contain videos that have different qualities, sizes, length, and color ranges, which indicates that our model is very adaptable and can be used with different video sources. Table 3 summarizes the datasets used in this work.

### 4.2. Experiments

In this section, we examine the performance of the proposed model while detecting and classifying videos as violent and nonviolent.

The experiments demonstrated the good performance of the proposed model, which has 4,074,435 parameters and is computationally light and relatively fast. Cross-validation was performed with five folds.

Averages of accuracy, precision, and F1 score were used to validate the performance of the proposed violence detection model. Accuracy and precision terms follow:

(1) Accuracy is the ratio of the correctly labeled samples to the whole set of samples. Accuracy is calculated as:

(2) $$\mathrm{Accuracy}\;=\frac{\mathrm{TP}\;+\;\mathrm{TN}}{\mathrm{TP}\;+\;\mathrm{TN}\;+\;\mathrm{FP}\;+\;\mathrm{FN}}\;$$

where TP—true positive, TN—true negative, FP—false positive, FN—false negative.

(2) Precision is the ratio of the correctly labeled “Violent videos” to the total number of labeled “Violent Videos”. Precision is calculated as:

(3) $$\mathrm{Precision}\;=\frac{\mathrm{TP}}{\mathrm{TP}\;+\;\mathrm{FP}}\;$$

where TP—true positive, FP—false positive.

(3) F1 score is the harmonic mean of precision and recall. F1 score is calculated as:

(4) $$F1\;\mathrm{score}\;=\frac{2\;\times \;\mathrm{precision}\;\times \;\mathrm{recall}}{\mathrm{precision}\;+\;\mathrm{recall}}\;$$

where recall is the ratio of the correctly labeled

“Violent Videos” to all the videos which are actually violent and calculated as:

(5) $$\mathrm{recall}=\frac{\mathrm{TP}}{\mathrm{TP}\;+\;\mathrm{FN}}\;$$

(4) Averages are calculated using Equation (6):

(6) $$\mathrm{average}\;=\overset{N}{\underset{i=0}{\sum }}\frac{{\mathrm{frame}}_{i}}{N}\;$$

Table 4 shows the averages of inference time, accuracy, precision, and F1 score of the proposed model, based on the dataset used.

The model was trained and accuracy evaluated on a PC using an RTX 3070 TI graphics card, Intel I7 10700K processor, and 16 GB of RAM. During testing, the system consumed ~315 watts as measured from the power outlet. The model performance on the edge devices was verified on an NVidia Jetson Xavier NX development board. The board was set to the 20 W six core power mode and achieved an inference time of 930 ms. The performance is sufficient to evaluate 1 s clips every second on this single board computer. However, it should be noted that running a model on a more capable desktop-grade GPU such as 3070 Ti allows it to serve 20 video streams simultaneously while consuming less power per video stream (15.75 Watts). A system serving multiple cameras can still be implemented on the edge while providing all the proposed benefits, such as reduction in response time and reduction in the network load. It should be taken into account that the most economical deployment depends greatly on the development of the upcoming hardware pricing and availability.

**Figure 7:**
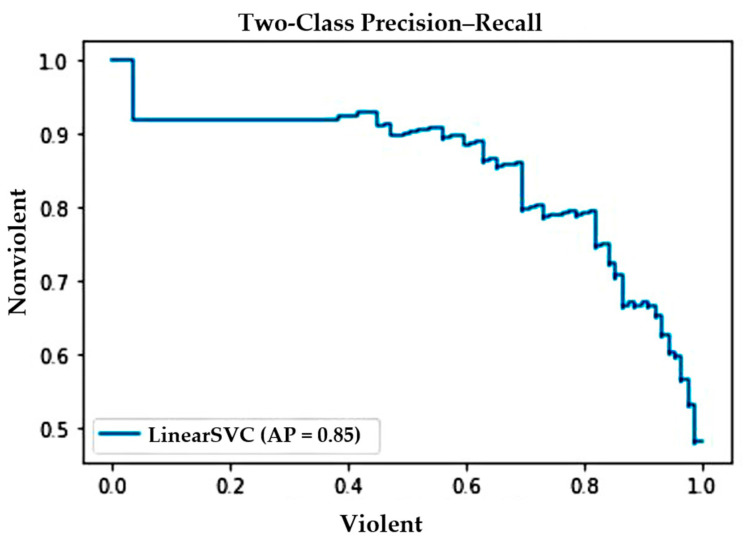
Precision–recall curve against RWF-2000 dataset.

The precision–recall curve shown in Figure 7 illustrates the tradeoff between precision and recall against the RWF-2000 dataset. In the figure, note that the curve represents a high score for recall and precision, which means the proposed method correctly labels the majority of results returned.

Table 5 compares the performance of other violence detection models tested with any of the following datasets: Hockey Fights, Movie Fights, and RWF-2000. When comparing our proposed method with those already existing, we can see that some perform better in terms of accuracy but are much heavier than ours. Generally, our method can easily compete in terms of accuracy. Best results were gained for the Hockey Fights and Movie Fights datasets where person-to-person violence was presented. The Movie Fights dataset was the least challenging owing to the nature of the video content.

Table 5 shows that our proposed model is more lightweight than previously proposed methods for violence detection. Although the models presented by Sudhakaran and Lanz [22], Akti et al. [42], and Rendón-Segador et al. [40] are slightly more accurate, our proposed model has a much lower count of parameters compared to these models, which makes our method faster and computationally efficient. The only model that has a lower number of parameters than ours was the end-to-end CNN-LSTM model presented by AlDahoul et al. [43]; however, experiments showed that this model is less accurate and less precise (model precision is 72.53 ± 4.6%) than ours.

In conclusion, the proposed model was tested using a complicated dataset collected in real-life and achieved promising results. Our model is lightweight and not computationally expensive, which is beneficial to use in time-sensitive applications or in edge devices.

## 5. Discussion

We present a model for violence detection, which is more efficient and more lightweight than previously presented methods. Experiments and model comparison is presented in the previous section of this paper. Despite the proposed model showing precise recognition of violent scenes, the test accuracy can be improved or the number of model parameters can be reduced. More tests using real-life datasets can be completed in the future because the Hockey Fights and Movie Fights datasets are not really effective as trainable examples for violent scenes; the violence in the videos does not exactly occur naturally. For future investigation, an interesting task would be to recognize action sequences that lead to the start of the violence.

Detecting violence in video data streams is very important for many applications. The proposed model demonstrates relatively high violence detection accuracy in three different datasets: RWF-2000, Movie Fights, and Hockey Fights. The latter two datasets consist of fighting scenes, which have common behavior. Violence usually starts at the moment when two persons slowly approach each other. There is much activity in the middle of the scene and again little or no motion can be detected at the end. These types of violence can be called synthetic because they lack unpredictability; they are recorded with great detail and all activity is captured in the center of the view. The RWF-2000 dataset consists of real-life scenes, which were recorded using video surveillance systems. In real-life videos, aggressive human behavior is a random event. Such an event can appear from nowhere in any location of camera view and can last from one second to several minutes. The violence can be more static (threatening with a gun) or very active (fist-fight). Therefore, we suggest that there is much space for improvement of the proposed violence detection method. First, our future work will involve the development of a well-balanced dataset with different video sources that are used in real-life surveillance systems. Additionally, the future model has to be capable of classifying the type of violence and not limited by one decision (is there any violence or not). In the future, combining features of several deep networks can lead us one step further in solving this difficult problem, such as crowd violence detection, development of algorithms that are suitable for UAVs (drones), and creation of classification methods that can be applied for conditions of limited annotated data.

Furthermore, exploring opportunities to expand the usability of our proposed system, we envisage that it can be adaptable for not only for the purpose it was created, but can also leverage the technologies of various sensor network implementations, thus improving the reaction time of human operators with larger systems inside of buildings and other complex structures (shopping centers, parking lots, airports, mines, factories) such as proposed by Wei et al. [47,48].

## 6. Conclusions

A novel and efficient method for detecting violent activities in real-life surveillance footage is presented in this paper. The proposed model is a spatial feature-extracting a U-Net like a network that uses MobileNet V2 as an encoder followed by LSTM for temporal feature extraction and classification. The model has 4,074,435 parameters. The architecture of the model makes it computationally light and fast. Cross-validation was performed with five folds, using three different datasets: Hockey Fights, Movie Fights, and RWF-2000. Using a complex real security camera footage dataset based on RWF-2000, experiments showed an average accuracy of 0.82 ± 2% and average precision of 0.81 ± 3%. The proposed model achieved good accuracy even though it is lightweight and not computationally expensive. Our model is beneficial to use in time-sensitive applications or in edge devices.

## Figures and Tables

**Table 1:** Summary of some related studies in violence detection.

Reference	Detection Methods	Feature Extraction	Strength
Gao et al. [31]	SVM and AdaBoost	Oriented violent flows (OViF)	Performance of the proposed OViF and LTP was able to achieve a more satisfactory accuracy of 87.50% and 88.00% for Hockey Fights and Violent Flow.
Peixoto et al. [32]	Inception v4, C3D, and a CNN-LSTM	Mel-frequency cepstral coefficients (MFCCs)	The proposed method was able to achieve an increasing accuracy of 6% in comparison with the baseline concept of violence with audio of 72.8% to 78.5% for both visual and audio features.
Accattoli et al. [33]	C3D + SVM	BoW or sparse coding information	Better detection rate—the proposed method is therefore general and can be applied in various scenarios
Zhou et al. [34]	SVM	Low-level features are the local histogram of oriented gradient (LHOG), bag-of-words (BoW), local histogram of optical flow (LHOF) descriptor	The proposed features extraction showed an effective detection model in automatic violent behaviors in comparison with the state-of-the-art algorithms.
Mohtavipour et al. [35]	CNN model	Differential motion energy image (DMEI) and DMOF	The proposed model was able to improve violence detection with an accuracy of approximately 100% for both crowded and uncrowded environments.

**Table 2:** A summary of some related studies in violence detection.

Layer	Output Shape	No. of Parameters #
Time distribution (U-Net features extractor)	(30, 64, 64, 1)	1,907,041
LSTM	(128)	2,163,200
Dense	(32)	4128
Dense	(2)	66
Total parameters: 4,074,435 Trainable parameters: 3,457,219 Nontrainable parameters: 617,216		

**Table 3:** Summary of datasets used.

Dataset	Size of Dataset	Framework Rate
RWF-2000	1600 videos	30 fps
Movie Fights	200 videos	25–30 fps
Hockey Fights	1000 videos	25 fps

**Table 4:** Summary of results based on the dataset.

Dataset	Avg. Inference Time, s	Avg. Accuracy, %	Avg. Precision, %	Avg. F1 Score
RWF-2000	0.046 ± 15%	82.0 ± 3%	81.2 ± 3%	0.782 ± 5%
Movie Fights	0.056 ± 10%	99.5 ± 2%	100 ± 0%	0.995 ± 2%
Hockey Fights	0.022 ± 2%	96.1 ± 1%	97.3 ± 2%	0.961 ± 1%

**Table 5:** Proposed model compared to existing studies in terms of accuracy and number of model parameters.

Method	Accuracy of RWF-2000 Dataset, %	Accuracy of Hockey Fights Dataset, %	Accuracy of Movie Fights Dataset, %	# of Parameters in the Model
ViolenceNet Optical Flow (Rendón-Segador et al. [40])	-	99.2 ± 0.6%	100 ± 0%	4.5 M
Efficient 3D CNN (Li et al. [41])	-	98.3 ± 0.81%	100 ± 0%	7.4 M
Xception + Bi-LSTM + attention for 5 frames (Akti et al. [42])	-	98 ± 0%	100 ± 0%	9 M
Xception + Bi-LSTM + attention for 10 Frames (Akti et al. [42])	-	97.5 ± 0%	100 ± 0%	9 M
ViolenceNet Pseudo-Optical Flow (Rendón-Segador et al. [40])	-	97.5 ± 1%	100 ± 0%	4.5 M
C3D (Tran et al. [28])	-	87.4 ± 1.2%	93.6 ± 1.2%	78.0 M
AlexNet + LSTM RNN (Sudhakaran and Lanz [22])	-	97.1 ± 3%	100 ± 0%	9.6 M
end-to-end CNN-LSTM (AlDahoul et al. [43])	73.35 ± 3%	-	-	1.266 M
Hough Forests + 2D CNN (Serrano et al. [44])	-	94.6 ± 0%	99 ± 0%	not specified
Three Streams LSTM (Dong et al. [45])	-	93.9 ± 0%	-	not specified
MoSIFT (Xu et al. [5])	-	93.6 ± 1.67%	-	not specified
Histograms of frequency-based motion intensities + AdaBoost (Deniz et al. [2])	-	90.1 ± 0%	98.9 ± 0%	not specified
ResNet50 + ConvLSTM (Sharma and Baghel [29])	-	89 ± 0%	92 ± 0%	not specified
Fine-tuned MobileNet model (Khan et al. [46])	-	87 ± 0%	99.5 ± 0%	not specified
Motion Blobs + Random Forest (Gracia et al. [8])	-	82.4 ± 0%	96.9 ± 0%	not specified
**Proposed model**	**82.0 ± 3%**	**96.1 ± 1%**	**99.5 ± 2%**	**4.074 M**

## Data Availability

Upon request from the authors.
